# Combinatorial Design of a Sialic Acid-Imprinted Binding Site

**DOI:** 10.1021/acsomega.1c01111

**Published:** 2021-04-29

**Authors:** Liliia Mavliutova, Elena Verduci, Sudhirkumar A. Shinde, Börje Sellergren

**Affiliations:** Department of Biomedical Sciences, Faculty of Health and Society, Malmö University, Malmö SE-20506, Sweden

## Abstract

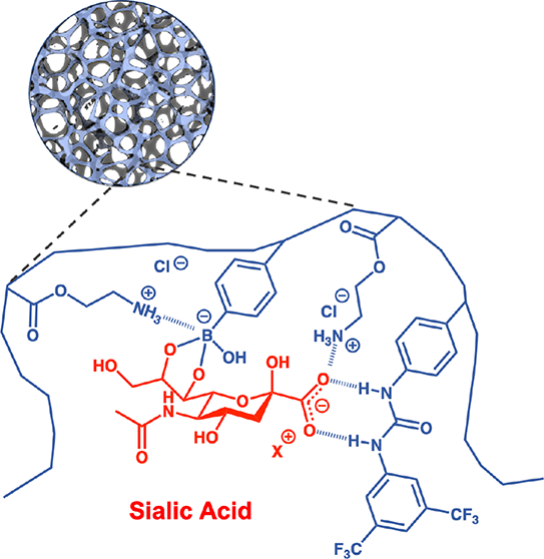

Aberrant glycosylation has been proven to correlate with various diseases including cancer. An important alteration in cancer progression is an increased level of sialylation, making sialic acid one of the key constituents in tumor-specific glycans and an interesting biomarker for a diversity of cancer types. Developing molecularly imprinted polymers (MIPs) with high affinity toward sialic acids is an important task that can help in early cancer diagnosis. In this work, the glycospecific MIPs are produced using cooperative covalent/noncovalent imprinting. We report here on the fundamental investigation of this termolecular imprinting approach. This comprises studies of the relative contribution of orthogonally interacting functional monomers and their synergetic behavior and the choice of different counterions on the molecular recognition properties for the sialylated targets. Combining three functional monomers targeting different functionalities on the template led to enhanced imprinting factors (IFs) and selectivities. This apparent cooperative effect was supported by ^1^H NMR and fluorescence titrations of monomers with templates or template analogs. Moreover, highlighting the role of the template counterion use of tetrabutylammonium (TBA) salt of sialic acid resulted in better imprinting than that of sodium salts supported by both in solution interaction studies and in MIP rebinding experiments. The glycospecific MIPs display high affinity for sialylated targets, with an overall low binding of other nontarget saccharides.

## Introduction

Sialic acids (SAs) are an important family of 9-carbon monosaccharides that are typically found as terminal moieties of *N*-glycans, *O*-glycans, and glycosphingolipids. SAs display incredible structural diversity with over 50 naturally occurring members, with *N*-acetylneuraminic acid (SA) being the most common mammalian SA.^1^ Sialic acids are involved in a variety of physiological and pathological processes, such as immune defense, cellular differentiation, cell–matrix interactions, and cell–cell adhesion.^2^ Many pathogenic bacteria and viruses utilize SA for avoiding immune response (e.g., group B Streptococcus) or for cell entry.^3^ Aberrant glycosylation is associated with a variety of diseases such as cardiovascular diseases, neurological disorders, and a diversity of cancer types. Generally, the total level of SAs in cancer is elevated, which is accompanied by changes in their modes of linkage.^4−7^

Lectins, naturally occurring carbohydrate-binding proteins, have monosaccharide affinities in the millimolar range. Their binding mostly relies on multivalency for increased affinities.^8^ Numerous SA-specific lectins are known, featuring specificity to certain types of glycosidic linkages.^9−11^ However, high cost, poor availability, low affinities in some cases and limitation in storage/application conditions limit the use of the lectins on a broader scale. The development of certain glycan-specific antibodies has been known to be particularly challenging because of glycan’s low immunogenicity and poor availability.^12^ Thus, the shortage of glycan-specific binders has been a major barrier for the advancement of glycan research.

The development of alternative glycan-specific receptors is of great importance for applications in glycomics, cell imaging/sorting, therapeutics, and drug development. Many synthetic receptors for glycan recognition are based on covalent boronate chemistry.^13−16^ It relies on the fast and reversible boronate bond formation between organic boronic acids and the diol functionalities on a sugar. Another group of carbohydrate receptors often utilizes strategically positioned hydrogen bonds in a relatively hydrophobic microenvironment to bind the guest. One successful example of such receptors, which involve noncovalent interactions with monosaccharides in aqueous media, is “temple” receptors developed by Davis and co-workers. These compounds contain aromatic residues as a “floor” and a “roof” and at least four relatively rigid “pillars” with appropriate hydrogen bond donor and acceptor groups.^17^ The syntheses of such complex receptors are quite cumbersome and limited to a number of saccharides, becoming too complex for larger targets. One alternative to overcome this problem is to use molecular imprinting. Here, a highly complementary binding site is formed by fixing preordered template/functional monomer interactions in a highly cross-linked polymer matrix. In this method, the synthetic route is much easier and faster to perform. Various molecular imprinting strategies for carbohydrate recognition have been reported, starting from early monosaccharide covalent boronate imprinting pioneered by Wulff^18^ followed by carbohydrate receptors targeting neutral mono/oligosaccharides^19^ and charged species such as SAs,^20^ hyaluronic acid,^21^ and glucuronic acid (GlcA).^21^ Impressive glycan recognition has been achieved with molecularly imprinted polymer (MIP) nanoparticles with oriented surface imprinting^22^ and tuned microenvironments^23^ and by employing reversible boronate interactions or hydrogen-bonded ion pairing to target saccharide diols or acid functionalities. Notwithstanding this important advance, applications of this technology to address real-world glycomics problems have not been reported. We previously introduced SA-imprinted fluorescent core–shell nanoparticles as a powerful tool for the selective labeling of cell surface glycans.^24,25^ Here, the glycospecific MIPs were produced using combined boronate-, amine-, and urea-based cooperative imprinting of SA. In this report, we have investigated in more detail the relative contribution of these monomers with the aim of developing practical MIP formats compatible with glycomics applications.

## Results and Discussion

**Figure 1:**
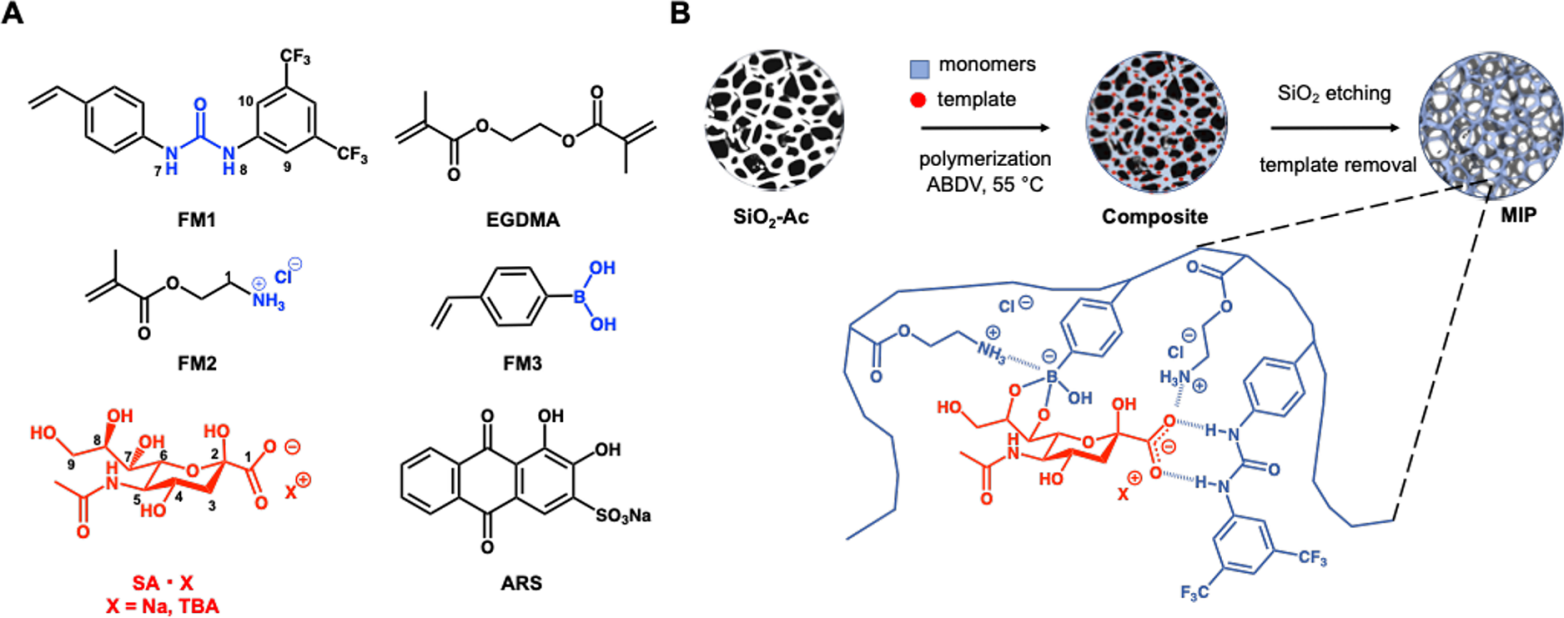
Structures of the monomers, SA template, and ARS (A) and silica-templated SA–MIP synthesis with the proposed configuration of the binding pocket (B).

With our previously reported SA-binding core–shell MIP as a starting point, fine-tuning of this design requires a comprehensive optimization of synthetic parameters and a better understanding of the underlying host/guest interactions. Our tightest binding host for SA incorporated three binding groups comprising a charge-neutral fluorescent urea-based, cationic ammonium-based (FM2), and boronic acid (FM3) monomers. The fluorescent urea-based monomer, used in the previous studies, was substituted with a nonfluorescent urea-based monomer (FM1) (Figure 1A). The rational was that these groups interact with the SA template, as exemplified in Figure 1B, resulting in a complementary binding site after polymerization. A cooperative effect of the monomers was supported by the results of the solubility experiments and spectroscopic characterization of the prepolymerization solution. 4-Vinylphenylboronic acid (FM3) targets C7–C8/C7–C9 diols or the α-hydroxy carboxylate functionality,^26^ whereas urea monomer FM1 likely engage in hydrogen bonds with the carboxylate group. The ammonium-based monomer (FM2) would serve as a Lewis base catalyzing the boronic acid esterification and in addition electrostatically stabilizes the carboxylate group. The resulting ternary or higher complexes would give rise to multifunctional binding sites with high avidity for the targeted glycan structure.^24^ To better predict the suitable host monomer and combination of monomers, we started by characterizing the monomer–template interactions in solution.

Focusing on optimizing the previously reported design, we chose to prepare macroporous polymers following conventional bulk or hierarchical imprinting procedures. This was also in line with our aim of implementing MIPs in glycomics. Optimization was primarily performed by changing one functional monomer or template at a time.

### ^1^H NMR Titration Studies

To verify monomer–template interactions, ^1^H NMR studies were conducted. Two functional monomers are used in this study—neutral urea (FM1) and charged amine-based (FM2) monomers. Two solvent systems, aprotic DMSO-*d*_6_ and protic CD_3_OD, and the effect of the template’s counterion on SA–monomer interactions were monitored. The latter parameter was deemed important since the choice of the counterion controls not only the template–monomer complex solubility but also the anion nakedness and hence the hydrogen bond interaction strength.

Hence, salts of SA with TBA^+^ and Na^+^ counterions were first formed to activate the carboxylate group to a varying degree for interaction with urea and amine monomers. The host monomer solutions (2 mM in DMSO-*d*_6_ or CD_3_OD) were titrated with a solution of the anion guest up to a 10-fold molar excess. Binding isotherms (Figures S1–S3) were fitted to a 1:1 Langmuir binding model to obtain the maximum complexation-induced shifts (CISs) and binding constants (*K*_a_) values, as summarized in Table 1. The urea protons (FM1) showed pronounced downfield shifts in the titrations performed in DMSO-*d*_6_, indicating the hydrogen bond formation between the host and guest molecules (Figure S1).

**Table 1:** Binding Constants and CISs for the Complexes between FM1 and FM2 with Different Salts of Sialic Acid in DMSO-*d*_6_ and CD_3_OD at 25 °C Obtained from Fitting to the Langmuir Monosite and Binary-Site Binding Model

host	guest	proton	DMSO-*d*_6_		CD_3_OD	
			*K*_a_, M^–1^	CIS, ppm	*K*_a_, M^–1^	CIS, ppm
FM1	SA·TBA	NH(7/8)	110 (±10)	4.073	a	0.025^b^
FM1	SA·Na	NH(7/8)	100 (±6)	3.699	a	0.015^b^
FM2	SA·TBA	CH2(1)	941 (±292)	0.020	c	c
			41 (±23)	0.053		
FM2	SA·Na	CH2(1)	843 (±393)	0.016	134 (±3)	0.010
			53 (±36)	0.036		

a Curve fitting precluded because of the lack of curvature.

b CIS at 10 equiv of host for CH(9/10).

c Biphasic curves showing a CIS minimum at a guest concentration of 3 mM.

However, the binding constants appear to be notably lower than those we previously reported for benzoate salts. For example, the binding constant of SA·TBA (*K*_a_ = 110 M^–1^) is nearly 2 orders of magnitude lower than the value reported for the TBA salt of benzoic acid (BA·TBA) (*K*_a_ = 8820 M^–1^). Nevertheless, this is in agreement with the results reported by Regueiro-Figueroa et al.^27^ and can be ascribed to its stronger acidity (*p*K_a_ ≈ 2.6 vs 4.2 for BA) and larger steric demand. As expected, only small shifts were observed in the protic solvent CD_3_OD, which is a stronger competitor for the hydrogen-bonding sites of the monomer and template.^24^ To be able to compare the two solvent systems, the shifts of the aryl protons CH(9/10) of FM1 were therefore used for curve fitting. However, as seen in Figure S2B, the CIS plots lacked strong curvature, especially for SA·TBA, which precluded any estimate of the binding constants. Nevertheless, the order of affinities was identical in both solvent systems with SA·TBA, resulting in higher CIS than SA·Na.

In the case of the amine monomer FM2, its HCl salt was used in the titration studies, and CH_2_(1) protons were used to construct the binding isotherm. Carrying a net +1 charge, this monomer was much less sensitive to the solvent switch and interacted strongly with both salts. In contrast to the isotherms of FM1, the curves indicated the presence of higher-order complexes and were best fitted with the Langmuir binary-site model. The presence of a steep initial slope and a clear inflection point at the 1:1 host/guest stoichiometry indicated binding constants of 941 M^–1^ for SA·TBA and 843 M^–1^ for SA·Na in DMSO-*d*_6_. In CD_3_OD, TBA·SA features an inflection point at 3 mM, indicating a complex binding behavior, whereas SA·Na fitted well to a mono-Langmuir binding model.

### Interaction between 4-Vinylphenylboronic Acid and a Fluorescent Diol

**Figure 2:**
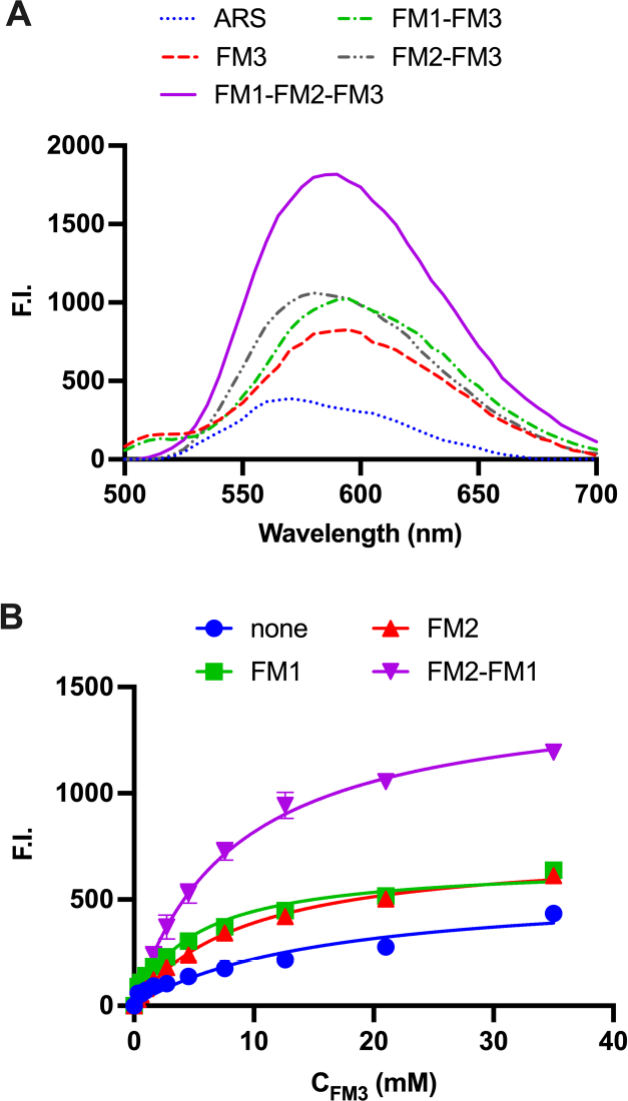
Fluorescence emission spectra of ARS (0.14 mM) in the presence of combinations of FM1, FM2, and FM3 (A) and fluorescence titration of ARS (0.14 mM) with FM3 at fixed concentrations of FM1, FM2, or mixture of FM1–FM2 (each 20 mM) (B). Spectra were recorded in methanol with λ_ex_/λ_em_ = 475/590 nm.

The cooperativity of the monomers was further confirmed by observing the interactions between alizarin red S (ARS) and the boronic acid monomer FM3 in the presence and absence of other comonomers. ARS is commonly used as a fluorescent reporter in carbohydrate–boronic acid interaction studies. Fluorescence of ARS increases drastically upon binding to boronic acids and therefore can be exploited as an indirect indicator of boronate–diol complexation strength. ARS is used here as a model diol instead of SA for its fluorescent properties. In this case, the results should be assessed cautiously because of different reactivities of ARS and SA. For example, equilibrium constants (*K*_eq_) are 1300 M^–1^ for ARS and 21 M^–1^ for SA with phenylboronic acid in 0.1 M phosphate buffer, pH 7.4.^28^ Nevertheless, it is an interesting method for ranking of the boronate–diol affinities and verifying complex stability. Thus, the interaction of ARS and FM3 in methanol was examined. Figure 2A shows the fluorescence emission spectra of ARS alone and in the presence of individual or combination of functional monomers. Interestingly, the maximum emission intensity depended strongly on the nature of the additive. While FM1 and FM2 alone or in combination led to fluorescence quenching, addition of the boronate monomer FM3 led to an enhanced emission intensity. This enhancement was slightly increased when FM1 or FM2 was added as the second monomer, indicating that these additives exerted only a minor influence on the ARS–FM3 interaction. This contrasted with the nearly 2-fold increase in the fluorescence intensity when all three functional monomers were added in combination. To verify that this was reflected in stronger interactions, we performed titrations of ARS with FM3. Figure 2B shows the binding curves obtained from the titrations of ARS with FM3 alone and in the presence of a fixed amount of FM1 or/and FM2 in methanol. Fitting the curves with the Langmuir monosite model resulted in the binding parameters shown in Table 2. The apparent binding constant (*K*_app_) of ARS–FM3 was 122 M^–1^ in the presence of 20 mM FM1 and FM2 and 64 M^–1^ in the absence of the comonomers. The addition of FM1 and FM2 alone resulted in *K* values of 200 and 109 M^–1^, respectively. Given the presence of higher-order complexes with a distribution that may change during the course of the titration, the *K* values as well as the degree of fluorescence enhancement are only indicative of the ARS–FM3 interaction strength. Nevertheless, the comonomers appear to assist the esterification in view of the 2- to 3-fold increase in the binding constants and 2-fold increase in the fluorescence intensity. We tentatively ascribe this cooperative effect to FM2-induced charge stabilization combined with excited state stabilizing urea–oxyanion interactions. A more detailed explanation of this effect and the mechanism and multiple equilibria of the system are beyond the scope of this study. For the time being, we can assume that it also strengthens the interactions between the SA template and boronic monomer as in the case of ARS. The displacement assay with SA did not yield reliable results. This could be due to a much higher affinity of ARS in the FM3–FM2–FM1 system. The maximum concentration of SA is also limited because of its limited solubility in methanol.

**Table 2:** Apparent Binding Constants and Fluorescence Intensity Maxima Obtained from the Titrations of ARS with FM3 Alone and in the Presence of Fixed Amount of FM1 or/and FM2 in Methanol

additive	*K*_app_ (M^–1^)	ΔFI	*R*^2^
	64 (±17)	562	0.9121
FM1	200 (±24)	668	0.9715
FM2	109 (±7)	749	0.9934
FM1 + FM2	122 (±9)	1490	0.9930

**Figure 3:**
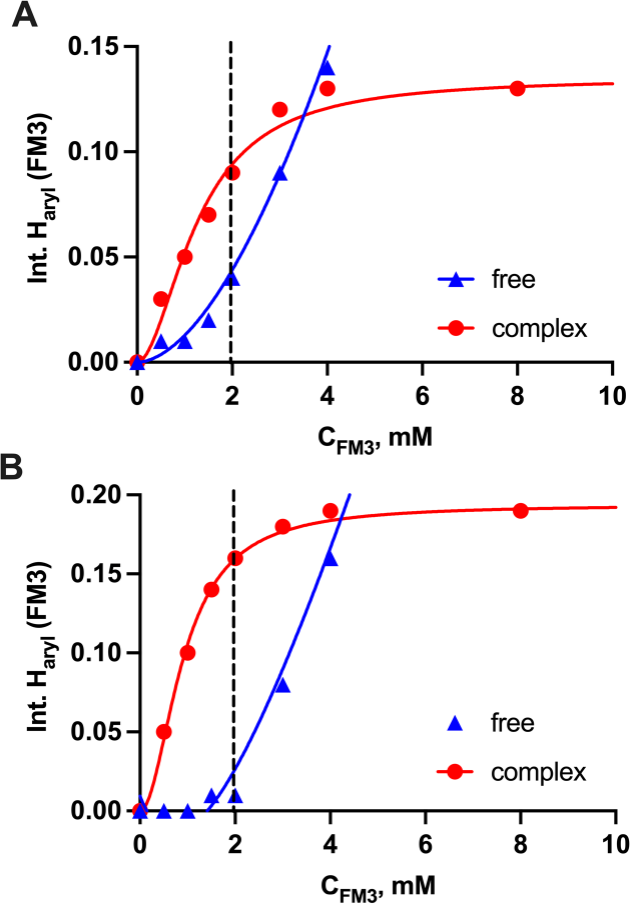
Integration of free and complexed aryl protons upon titration of 2 mM SA·TBA with FM3 in the absence (A) and presence (B) of 2 mM FM1 in DMSO-*d*_6_. The dashed line represents 1:1 equiv of FM3:SA·TBA.

To further corroborate these observations, we performed an ^1^H NMR titration of FM1 with SA·TBA in the presence of 1 equiv of FM3 and 0–2 equiv of FM2 to mimic the prepolymerization stoichiometry. Titration of FM1 + FM3 with SA·TBA resulted in a curve with an inflection point at 1 equiv of the guest, indicating the presence of a stable higher-order complex (Figure S4A). This is notably absent in the titration of FM1 with SA·TBA alone (Figure S1). Addition of FM2 to the guest system led to a shallower curve but still featuring the 1:1 inflection point (Figure S4B). The multiple solution equilibria complicate any modeling of the CIS plots. However, the esterification of FM3 with SA appears to induce a tight interaction site for FM1. Moreover, titrations of FM3 and FM3 + FM1 with SA·TBA and vice versa of SA·TBA ± FM1 with FM3 in DMSO-*d*_6_ support boronate complex formation (Figures S5–S8). In the former case (Figures S5 and S6), the signal of the −B(OH)_2_ group at 8.05 ppm disappears upon increasing the amount of SA, whereas the signals from the aromatic protons of the FM3–SA complex at 7.2 and 7.35 ppm increase. Performing the same titration in the presence of FM1 led to slightly different results. The gradual downfield shift of the FM1 aryl proton at 8.13 ppm as well as the shift and splitting of the −B(OH)_2_ signal in addition to the expected increase in the signals at 7.20 and 7.35 ppm (complexed FM3) collectively supports the involvement of both monomers in the complex. Titrations of SA·TBA and SA·TBA + FM1 with FM3 are shown in Figures S7 and S8, respectively. The appearance of the signals corresponding to free FM3 at 7.42 and 7.75 ppm in addition to the signals from complexed FM3 indicates incomplete complex formation when titrating SA·TBA alone. Interestingly, in the presence of FM1, the signals from free FM3 are completely absent again, supporting cooperativity between FM3 and FM1 in complexing SA·TBA. The results are summarized in Figure 3. Higher concentrations of the monomer–SA·Na combinations in DMSO-*d*_6_ and CD_3_OD yielded similar results, indicating the presence of the boronate complexes (Figure S9).

### Study of Functional Monomer Cooperativity

In view of the relatively weak interactions measured for the individual functional monomer–diol pairs, we decided to first confirm whether the cooperative behavior persisted under the conditions used in the molecular imprinting step. Thus, the conventional solution polymerized “bulk” polymers were prepared using SA·TBA as a template (T), ethylene glycol dimethacrylate (EGDMA) as a cross-linker, methanol as a porogen, and *N*,*N*′-azo-bis(2,4-dimethyl)valeronitrile (ABDV) as a thermal initiator. A library of imprinted polymers **P1**–**P7** was prepared using the molar ratios of the template monomers FM1:FM2:FM3:EGDMA:T, as stated in Table 3. Nonimprinted polymers (NIPs) **P_N_1–P_N_7** were prepared similar to MIPs, but with the omission of template addition. The polymers were crushed and sieved to obtain 25–50 μm particles with subsequent template removal by acidic solvent extraction.

**Table 3:** Composition of SA·TBA Imprinted Polymers (**P1**–**P7**)^a^ with the Molar Ratios of the Monomers and Template

MIP	FM1	FM2	FM3	EGDMA	T
**P1**	0	2	0	20	1
**P2**	0	0	1	20	1
**P3**	1	0	0	20	1
**P4**	0	2	1	20	1
**P5**	1	2	0	20	1
**P6**	1	0	1	20	1
**P7**	1	2	1	20	1

a NIPs **P_N_1**–**P_N_7** were produced in a similar way with the omission of the template addition.

**Figure 4:**
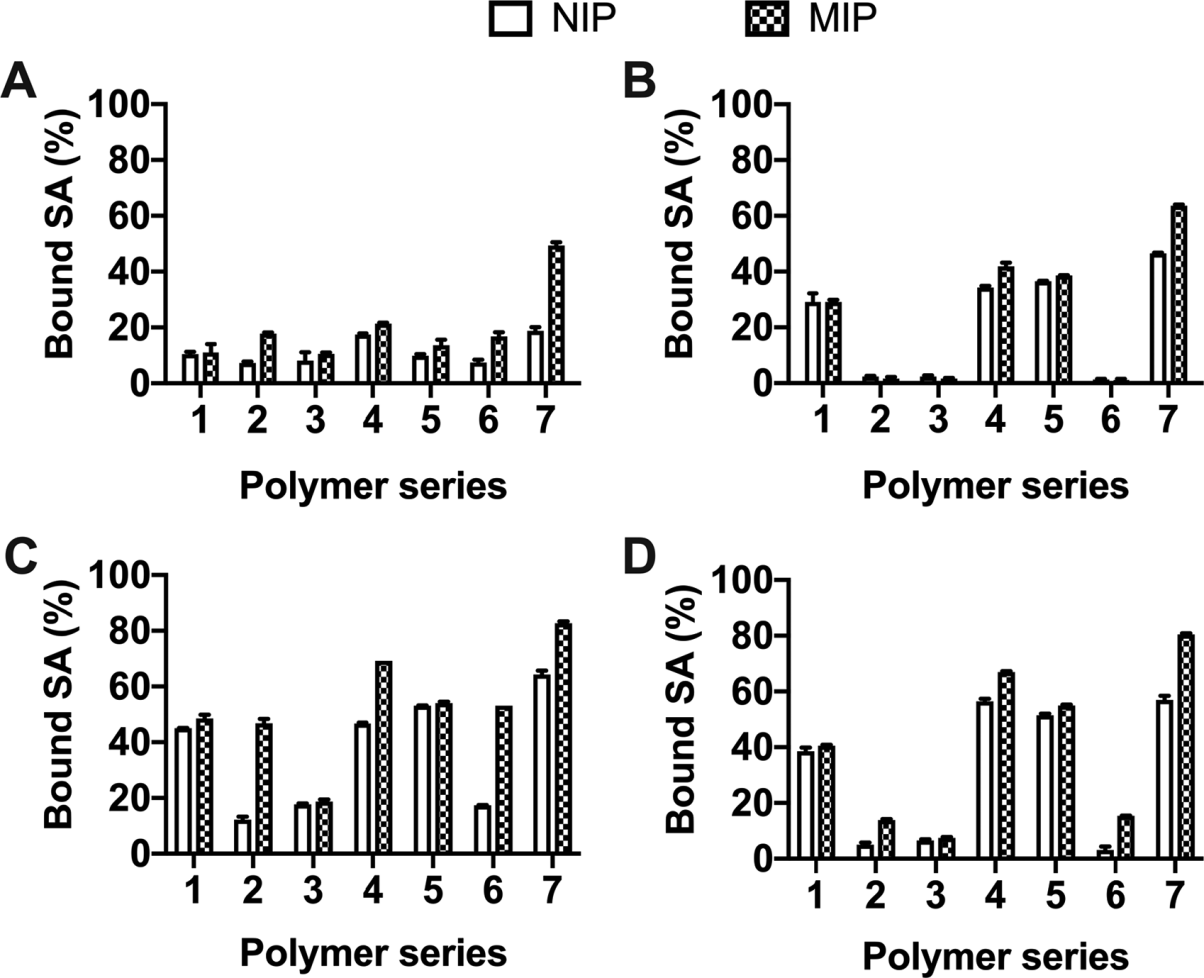
Binding of 0.5 mM SA in 100% (A) and 10% (B) MeOH and SA·TBA in 100% (C) and 10% (D) MeOH by the library of MIPs (**P1**–**P7**) and NIPs (**P_N_1**–**P_N_7**). The average of three replicas is shown with error bars representing standard deviation.

MIP and NIP materials were then assessed for their template rebinding behavior. Batch binding tests were performed with 0.5 mM SA in its free acid form and as TBA salt in 100% and 10% MeOH. This solvent was used in the polymerization step and expected to promote optimal polymer chain conformation, thus enhancing imprinting efficiency. The results of the rebinding experiment are shown in Figure 4. When monomers FM1, FM2, and FM3 were used separately to produce MIPs, the imprinting efficiency was low. However, strong cooperativity could be seen when all three monomers were used for the SA imprinting (**P7**/**P_N_7**), resulting in the highest binding capacity and IF (2.6) among all permutations (Figure 4A). In water-rich media, binding of polymers composed only of boronate (**P2**/**P_N_2**) and urea-based (**P3**/**P_N_3**) monomers, as well as their combination **P6**/**P_N_6**, was negligible (Figure 4B). This is explained by the disruption of hydrogen bonds between urea and the template and the lack of boronate esterification in the presence of water at low pH. However, FM2-based polymers exhibit a dramatic increase in SA uptake going from organic to aqueous media. Thus, the addition of the amine monomer plays a crucial role in enhancing their capacity in an aqueous environment. Although the reference polymer **P_N_7** also displays significant uptake, there is no obvious correlation with the extent of nonspecific binding to **P7**. The reactivity ratios of charged monomers strongly depend on counterions and solvent, suggesting that **P7** and **P_N_7** may feature entirely different structures and microenvironments (vide infra). When changing to the TBA salt of SA, the binding capacities in both methanol and water-rich media generally increased (Figure 4C,D). Here, an elevated imprinting efficiency for polymers **P2** and **P6** was observed, with IF = 3.8 and 3.1, respectively, albeit with binding suppressed in the water-rich solvent system. A combination of all three functional monomers still yields the highest binding capacity, with an almost 2-fold increase going from SA to SA·TBA. Since the MIPs were produced using SA·TBA as a template, the counterion memory effect can here play a role.^29^ TBA also acts as an activator for the carboxylic group on SA and a phase-transfer agent.

### Hierarchically Imprinted Polymers

Our next goal was to verify whether the aforementioned polymerization protocol could be transferred to formats applicable in glycomics/proteomics. For this purpose, we assessed our previously reported controlled pore size approach based on the silica templating technique.^30^ The polymers were prepared as outlined in Figure 1 using mesoporous (*D*_p_ ≈ 50 nm) spherical silica microparticles as vessels for in-pore polymerization. Acetylated silica was allowed to soak in the prepolymerization mixture and then thermally cured at 55 °C. After 24 h, the silica mold was dissolved by treatment with an aqueous solution of NH_4_HF_2_, leaving behind organic polymer particles, with size and morphology reflecting those of the original silica beads. The polymers were characterized by thermal gravimetric analysis (TGA), scanning electron microscopy (SEM), elemental analysis (EA), and IR spectroscopy (Tables S1 and S2, Figures S10 and S11). FTIR and EA confirmed their identity and near-identical chemical compositions, whereas TGA confirmed a quantitative removal of the silica template. SEM images furthermore confirmed that the silica-templated materials retained the spherical shape and size of the silica scaffold after etching. This proves that the beads correspond to the polymer formed in the silica pores.

**Figure 5:**
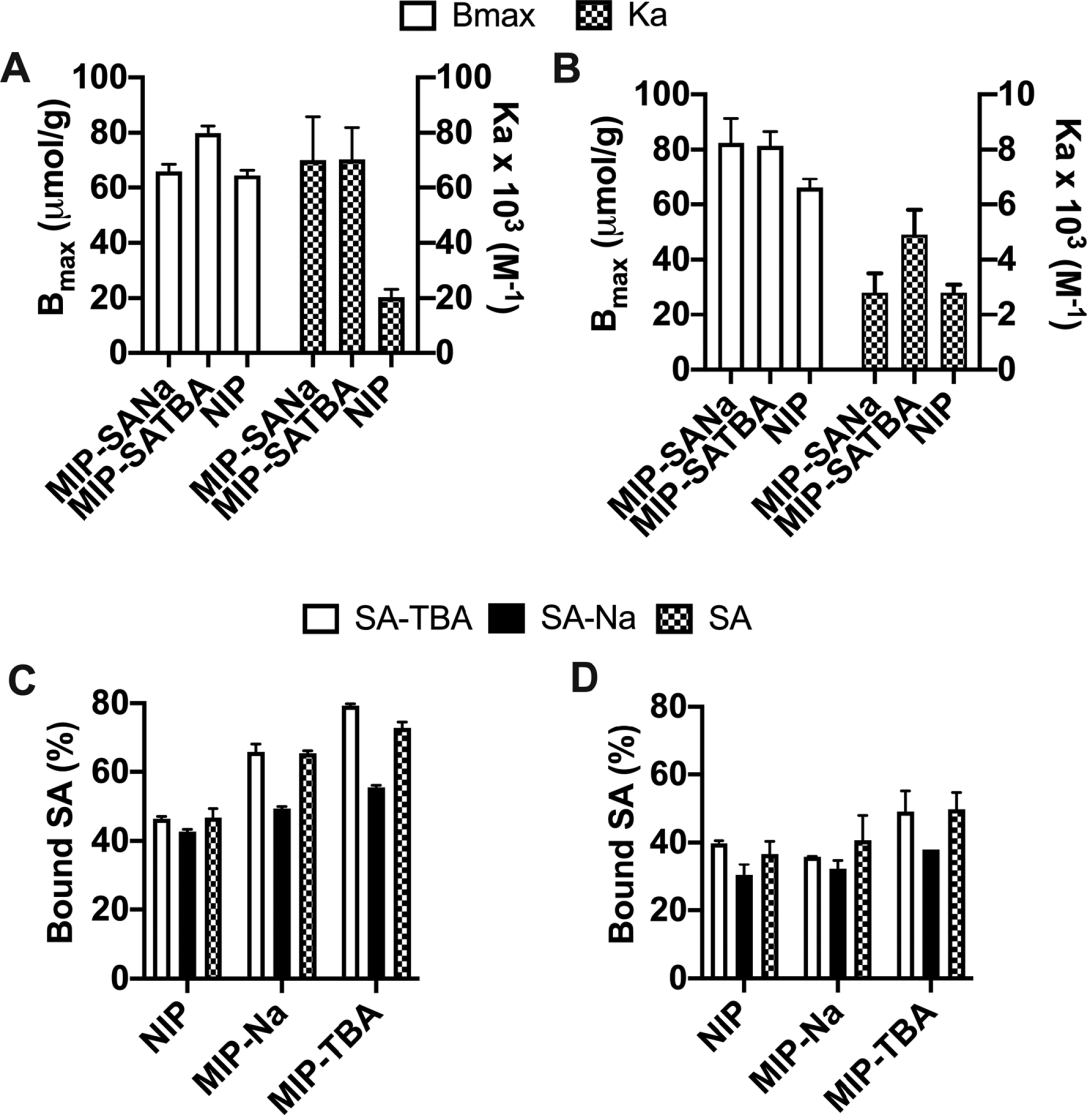
Binding parameters *B*_max_ and *K*_a_ in 100% (A) and 10% (B) MeOH obtained from the binding isotherms of SA with SA·TBA- and SA·Na-imprinted polymers and NIP. Uptake of 1 mM SA, SA·Na, and SA·TBA in 100% MeOH (C) and 10% MeOH (D) with SA·TBA- and SA·Na-imprinted polymers and NIP. The average of three replicas is shown with error bars representing standard deviation.

With the aim of verifying the counterion effects observed in the solution complexation studies, different salts of SA were used as templates. Hence, polymer beads were prepared with TBA^+^ and Na^+^ salts of SA using ratios of monomers and template as SA:FM1:FM2:FM3:EGDMA = 1:1:2:1:20. To compare the affinity and capacity of the materials, we constructed the binding isotherms of all materials for the SA in 10% and 100% MeOH (Figure S12). The binding capacity (*B*_max_) and binding constants (*K*_a_) are summarized in Figure 5A,B. The overall imprinting efficiency was slightly lower for the hierarchically imprinted polymers compared to the bulk or to our previously reported core–shell polymers (*K* = 6.6 × 10^5^ M^–1^ in 98% MeOH for SA and 3.3 × 10^4^ M^–1^ for GlcA).^24^ The polymer imprinted with the TBA^+^ salt showed a higher capacity than when Na^+^ was used as a counterion, with a slight preference for the template salt (Figure 5C,D). This is again indicative of a weak template memory effect and agrees with the results from the monomer–template complexation study. The overall high uptake of SA·TBA is attributed to the hydrophobic butyl chains of this agent, promoting the transfer of the highly polar hydrophilic template into the hydrophobic polymer network.

### MIP Selectivity for Monosaccharides

**Figure 6:**
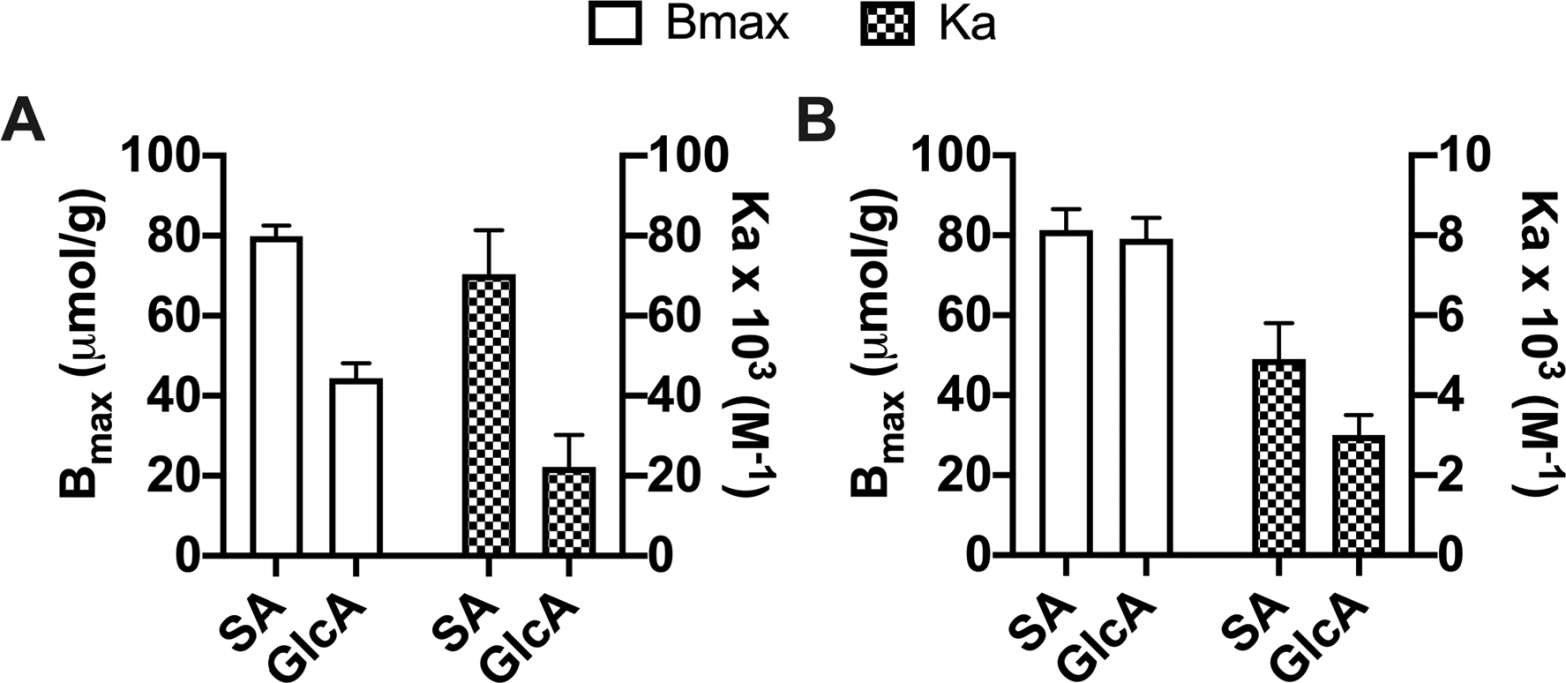
Binding parameters *B*_max_ and *K*_a_ in 100% (A) and 10% (B) MeOH obtained from the binding isotherms of SA and GlcA with MIP–SA·TBA. The average of two replicas is shown with error bars representing standard deviation.

The recognition performance of the materials is confirmed by the binding isotherms shown in Figure 6. GlcA serves as a reference anion for determining the selectivity of the imprinted material. SA and GlcA are acid sugars with similar dissociation constants (*p*K_a_ ≈ 2.6^31^ and 3.0,^32^ respectively) and propensity for binding to phenylboronic acid, with SA featuring a *K*_a_ = 21 M^–1^ and GlcA featuring a *K*_a_ = 16 M^–1^ in 0.1 M phosphate buffer, pH 7.4.^28^ Binding experiments were conducted in 100% and 10% methanol using MIP–SA·TBA. Data from the curve fitting are shown in Figure 6 (Tables S3 and S4, Figure S13). The MIP binding affinity for SA (*K*_a_ = 70 × 10^3^ M^–1^) is significantly higher than that observed for the GlcA (22 × 10^3^ M^–1^), a nontarget of the MIP. In agreement with our previous report,^24^ MIP performance deteriorates in water-rich media but still retains its preference for the template SA over GlcA. This confirms the hierarchical imprinting format as being compatible with the imprinting of simple saccharides.

**Figure 7:**
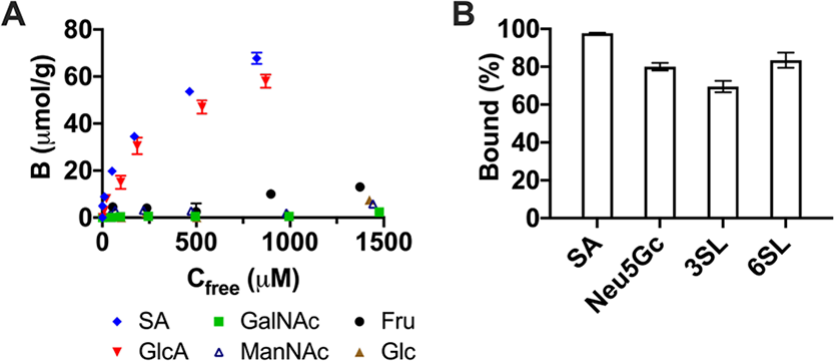
Monosaccharide binding in 10% MeOH (A) and uptake of sialylated targets (0.5 mM) in 100% MeOH (B) by MIP–SA·TBA. The average of two replicas is shown with error bars representing standard deviation.

The cross reactivity with different common monosaccharides was thereafter investigated (Figure 7A). The binding of neutral saccharides such as glucose (Glc), fructose (Fru), *N*-acetyl-d-mannosamine (ManNac), and *N*-acetyl-d-galactosamine (GalNAc) in 10% MeOH was low. Within this group, Fru showed the highest binding, in agreement with its overall high affinity toward boronic acids. Hence, the main contribution to the binding comes from ionic interactions involving sialic acid and GlcA and FM2 assisted by the boronate affinity promoted by FM3. High uptake of GlcA might be attributed to its relatively small size in comparison with SA, its similar *p*K_a_ values, and similar boronate affinity (vide supra). Binding of different forms of SAs, such as SA and Neu5Gc, as well as sialylated trisaccharides 3SL and 6SL is shown in Figure 7B.

A glycosidic bond at the C2 position of the SA lowers the overall uptake as seen in the case of 3SL and 6SL binding. This could be attributed to the effect of α- and β-anomers. Free SA typically exists in solution in its major β-form. All biological sialylated targets, however, feature α-glycosidic linkages. Nevertheless, the preference for SA versus Neu5Gc forms of SAs is apparent, with the difference between both sugars being only in one hydroxyl group.

## Conclusions

Expanding on our previous report on fluorescent probes for cell surface sialylated glycans, our aim here has been to dissect the site construction to identify key contributions to the molecular recognition performance. A study of the influence of orthogonally interacting functional monomers clearly shows that potent binding sites can be constructed, exploring cooperatively acting monomers. This effect was confirmed by both solution complex formation studies and by preparation and characterization of a small MIP combinatorial library. Moreover, the work has demonstrated the importance of a correct choice of template counterions. Choosing counterions promoting carboxylate hydrogen bond acceptor strength leads to increased binding affinity in homogenous solution as well as heterogeneous polymer–solution systems. Studies are ongoing to exploit this effect further. Finally, we have shown that silica-templated MIP synthesis can be combined with monosaccharide imprinting to yield controlled pore and bead-size materials. Given the tunable pore size, this class of materials is compatible with peptide separations as in proteomics and glycomics. Further optimization of the synthesis parameters is in progress to adapt the materials to glycomics workflows.

## Experimental Section

### Materials

ABDV was from Wako Chemicals GmbH (Neuss, Germany). EGDMA and ARS were from Acros Organics. 4-Vinylphenylboronic acid (FM3), ammonium hydrogen difluoride (NH_4_HF_2_), acetic acid, acetic anhydride, phenol, ammonium acetate, formic acid, dimethylformamide (DMF), dry methanol (MeOH), DMSO-*d*_6_, and methanol-d_4_ (CD_3_OD) were from VWR chemicals. All solvents for high-performance liquid chromatography (HPLC) analysis were of HPLC grade and were purchased from VWR. 2-Aminoethyl methacrylate hydrochloride (FM2) was received from Polysciences. Amino-functionalized macroporous silica beads (NH_2_@SiO_2_) with an average particle size of 30 μm, a surface area (S) of 45 m^2^g^–1^, an average pore diameter (*D*_p_) of 47.5 nm, and a pore volume (*V*_p_) of 0.81 mL/g were purchased from Fuji Silysia Chemical Ltd. (Kozojicho, Kasugai Aichi, Japan). Monosaccharides Glc, galactose (Gal), Fru, and TBA hydroxide (TBA–OH) 1 M in methanol were obtained from Sigma-Aldrich. *D*-GlcA was received from Fluka. *N*-Acetylneuraminic acid, *N*-glycolylneuraminic acid (Neu5Gc), GalNAc, ManNac, 2,6′-sialyllactose sodium salt (6SL), and 2,3′-sialyllactose sodium salt (3SL) were purchased from Carbosynth Ltd. (UK).

EGDMA was passed through a column of activated basic alumina to remove the inhibitor and stored at −20 °C before polymerization. *N*-3,5-Bis(trifluoromethyl)-phenyl-*N*′-4-vinylphenylurea (FM1) was synthesized as previously reported.^33^

### Instrumentation

^1^H NMR spectra were recorded using an Agilent Mercury 400 MHz instrument. HPLC measurements were carried out on an Alliance 2795 instrument equipped with a 2996 PDA detector (Waters, Milford, MA, USA). Polymer morphology and size were determined using a Zeiss EVO LS 10 (E) SEM (Carl Zeiss AG, Oberkochen, Germany). Infrared spectra were recorded using a Thermo Nicolet Nexus 6700 instrument (Thermo Scientific, Waltham, MA, USA). UV absorbance and fluorescence measurements were performed on a Safire plate reader (Tecan Group Ltd., Männedorf, Switzerland) using a polystyrene 96-well microplate. Elemental analysis was performed at Nicolaus Copernicus University in Toruń on a Vario MACRO 0000 (Elementar Analysensysteme GmbH, Germany). Elemental analysis was performed at the Department of Organic Chemistry, Johannes Gutenberg Universitat Mainz using a Heraeus CHN-rapid analyzer (Hanau, Germany).

### Sialic Acid Salt Preparation

Salts of SA were prepared by adding equimolar amounts of NaOH or TBA–OH to SA in methanol and evaporating the solvent in vacuo, producing SA·Na or SA·TBA, respectively.

### ^1^H NMR Titrations

Increasing amount of guest (SA·TBA or SA·Na) was titrated into a constant amount of functional monomers in DMSO-*d*_6_ or CD_3_OD. The concentration of the functional monomer was 2 mM, and the amount of the added guest was 0, 0.25, 0.5, 0.75, 1.0, 1.5, 2.0, 4.0, 6.0, and 10.0 equiv. The CISs of relevant protons were followed, and titration curves of CIS versus free guest concentration (*C*) were constructed. The raw titration data were fitted to the 1:1 binding site model (eq 1):
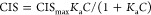
1where CIS_max_ is the maximum CIS at saturation and *K*_a_ is the binding constant.

The fitting was performed by nonlinear regression using GraphPad Prism version 9.0 (GraphPad Software, La Jolla, CA, USA).

Increasing amount of FM3 was titrated into a constant amount of SA·TBA (2 mM) and SA·TBA + FM1 (2 mM:2 mM) in DMSO-*d*_6_. ^1^H NMR spectra of monomer–template combinations were recorded in CD_3_OD and DMSO-*d*_6_ with 20 mM SA·Na.

### ARS Fluorescence and Absorbance Studies

Absorbance (350–600 nm) and fluorescence spectra (500–700 nm, λ_ex_ = 475 nm) of ARS (0.14 mM) in the presence of various combinations of FM1, FM2, and FM3 (each 20 mM) in methanol were recorded in a 96-well plate with 100 μL of solutions. ARS (0.14 mM) was titrated with 0.01–30 mM FM3 in methanol, with and without 20 mM FM1/FM2. The fluorescence intensity of the complex was measured (λ_ex_ = 475/590 nm).

### Bulk Polymer Synthesis

The following general procedure was used for preparing SA-imprinted polymers **P1**–**P7**. Template SA·TBA (0.1 mmol), functional monomers FM1 (0.1 mmol), FM2 (0.2 mmol), FM3 (0.1 mmol), and EGDMA (2 mmol) were dissolved in 1 mL of dry methanol. The initiator ABDV (1% wt/wt of total monomers) was added to the solution. The solution was cooled to 0 °C and purged with a flow of dry nitrogen for 10 min. The polymerization was initiated by placing the tubes in a water bath heated to 55 °C. After 24 h, the polymers were lightly crushed; washed with 1 × 10 mL MeOH, 4 × 10 mL MeOH/0.1 M HCl (80:20 v/v), 4 × 10 mL MeOH/H_2_O (80:20), and 2 × 10 mL methanol; and finally dried in vacuo. The wash fractions were analyzed by LC-MS and phenol–sulfuric assay.^34^ NIPs (**P_N_1**–**P_N_7**) were prepared in the same manner as described above but with the omission of the template from the prepolymerization solution.

### Silica End-Capping

NH_2_@SiO_2_ (20 g) was suspended in 100 mL of DMF in a 250 mL round-bottom flask. Next, 20 mL of acetic anhydride was added, and the suspension was stirred at room temperature (RT) overnight. Thereafter, the silica was filtered off, washed with DMF (3 × 50 mL) and MeOH (3 × 50 mL), and dried in vacuum overnight to yield *N*-acetylated silica (Ac@SiO_2_). Successful functionalization was confirmed by the ninhydrin test and TGA analysis.

### Silica-Templated Polymers

SA·Na and SA·TBA were used as SA templates to produce MIP–SA·Na and MIP–SA·TBA. The prepolymerization mixture was prepared by dissolving templates (30.9 mg, 0.1 mmol) in 1 mL of dry methanol, followed by the addition of FM3 (14.8 mg, 0.1 mmol), FM1 (37.4 mg, 0.1 mmol), FM2 (33.1 mg, 0.2 mmol), and the cross-linker EGDMA (396.4 mg, 2 mmol). The mixture was cooled on an ice bath while being purged with N_2_ for 10 min, followed by the addition of ABDV (1% wt/wt of total monomers). NIPs were prepared in a similar manner but omitting the addition of templates. The samples of Ac@SiO_2_ (1.25 g) were first deaerated in 50 mL Schlenk tubes (three-cycle vacuum-N_2_ purge) and then allowed to soak in prepolymerization mixtures (SA–MIP and NIP) (0.75 ml) under a nitrogen atmosphere. Next, the tubes were sealed and placed in a water bath heated to 55 °C to polymerize. After 24 h, the resulting composite beads were transferred to 50 mL polypropylene centrifugation tubes followed by the addition of the etching solution (3 M NH_4_HF_2_, aqueous). The tubes were shaken on a rocking table for 24 h. Thereafter, the polymer beads were washed with water, MeOH/0.1 M HCl (80:20 v/v) (3 × 50 mL), and MeOH (3 × 50 ml). The resulting polymers (SA–MIP and NIP) were dried in vacuo overnight.

### Equilibrium Binding Tests

Polymers (5 mg each) were suspended in 0.5 mL of 0.5 or 1 mM SA, SA·Na, SA·TBA, 6SL/3SL, Neu5Gc solution in 100% or 10% MeOH, and shaken for 24 h. Afterward, the samples were centrifuged and the supernatant (0.2 mL) was dried (Genevac EZ-2 evaporator), redissolved in 0.2 mL of mobile phase, and analyzed by HPLC-UV in the HILIC mode using a PolyHYDROXYETHYL A column (PolyLC Inc., 3 μm, 100 Å, 100 × 3.2 mm). Mobile phases were (A) acetonitrile and (B) ammonium acetate buffer (10 mM, pH = 5.0). An isocratic method of 75% A and 25% B at a flow rate of 0.5 mL/min was used. The injection volume was 10 μL, and the detection was performed by UV absorbance measurements at 205 nm. Each experiment was performed in triplicate. The resulting peak areas were used to calculate the binding capacity of the polymer (*B*) according to eq (2):
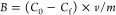
2where *C*_0_ is the initial solute concentration, *C*_f_ is the final solute concentration in the supernatant, *v* is the total volume of the adsorption mixture, and *m* is the mass of polymer.

IF was calculated according to eq (3):
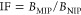
3where *B* equates to the binding capacity of MIP and NIP.

### Phenol–Sulfuric Assay

The phenol–sulfuric colorimetric assay was used to measure carbohydrate concentrations.^34^ First, 25 μL of 5% wt/wt phenol was added to 25 μL of aqueous carbohydrate analyte solution previously aliquoted into the microplate and mixed with a pipettor. Next, 150 μL of H_2_SO_4_ was added to each well and mixed with a pipettor. The solutions were incubated for 15 min at 80 °C. After cooling to RT, the absorbance was read at 490 nm in the microplate reader.

### Binding Isotherms

Polymers (5 mg each) were separately mixed with 0.5 mL of saccharides at 0.05, 0.1, 0.25, 0.5, 1.0, and 1.5 mM concentrations in 100% or 10% MeOH and shaken for 24 h at RT. Next, the samples were centrifuged, and the supernatant was analyzed either by HPLC-UV (for SA, GalNAc, ManNAc) or phenol–sulfuric assay (Glc, Fru, GlcA) using the methods described above to determine the concentration of unbound saccharides. The amount of bound saccharide per unit mass of polymer (*B*) was calculated according to eq 2. Each experiment was performed in duplicate. Binding curves were constructed by plotting *B* against free concentration *C_f_* and were subsequently fitted by nonlinear regression in the GraphPad Prism software (GraphPad, USA) to a Langmuir monosite model (eq 4):
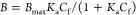
4where *B*_max_ is the maximum amount of solute bound by the polymer particles at saturation and *K*_a_ is the binding constant.

## Supporting Information Available

^1^H NMR titration curves, polymer characterization by TGA, EA, FTIR, SEM, and binding isotherms (PDF)
