# Supplementary material for: Combinatorial Design of a Sialic Acid-Imprinted Binding Site
Source: ACS Omega. 2021 Apr 29;6(18):12229–37. doi: 10.1021/acsomega.1c01111 (PMC8154165; doi:10.1021/acsomega.1c01111)
Supplement: Supplementary file 1 — ao1c01111_si_001.pdf [file ao1c01111_si_001.pdf]

Supporting Information

Combinatorial design of a sialic acid imprinted binding site

Liliia Mavliutova, Elena Verduci, Sudhirkumar A. Shinde,† Börje Sellergren*

Department of Biomedical Sciences, Faculty of Health and Society, Malmö University, SE-20506 Malmö, Sweden.

† School of Consciousness, Dr Vishwanath Karad Maharashtra Institute of Technology – World Peace University, Kothrud, Pune 411038, India

Supplementary Tables

Table S1. TGA weight loss for various silica templated polymers.

Polymer	Weight loss, %
**SATBA-MIP**	96.9
**SANa- MIP**	96.5
**NIP**	97.1
**$NH_2@SiO_2$**	2.2
**$Ac@SiO_2$**	3.1
**$Ac@SiO_2$-MIPTBA**	29.7

Table S2. Elemental analysis of silica templated polymers SATBA-MIP and NIP

Name	N [%]	C [%]	H [%]	C/N
**SATBA-MIP**	1.41	58.32	7.214	41.42
**NIP**	1.40	59.67	7.59	42.53
**Theoretical**	1.29	58.82	6.81	45.59

Table S3. Values from fitting to one site specific (Langmuir mono-site isotherm) from binding SATBA-MIP/NIP in 100%MeOH

Saccharide	Sialic acid		Glucuronic acid	
	SA-MIP	NIP	SA-MIP	NIP
**B_{max}, umol/g**	80 ±3	65 ± 2	44 ± 4	43 ± 6
**K_a x 10^3, M^{-1}**	70 ± 11	20 ± 3	22 ± 8	45 ± 2
**R^2**	0.9814	0.9867	0.8788	0.8508

Table S4. Values from fitting to one site specific (Langmuir mono-site isotherm) from binding SATBA-MIP/NIP in in 10%MeOH

Saccharide	Sialic acid		Glucuronic acid	
	SA-MIP	NIP	SA-MIP	NIP
**B_{max}, umol/g**	81 ± 5	66 ± 3	79 ± 5	63 ± 6
**K_a x 10^3, M^{-1}**	4.9 ± 0.9	2.8 ± 0.3	3.0 ± 0.5	4.1 ± 1.2
**R^2**	0.9793	0.9913	0.9850	0.9442

Supplementary Figures

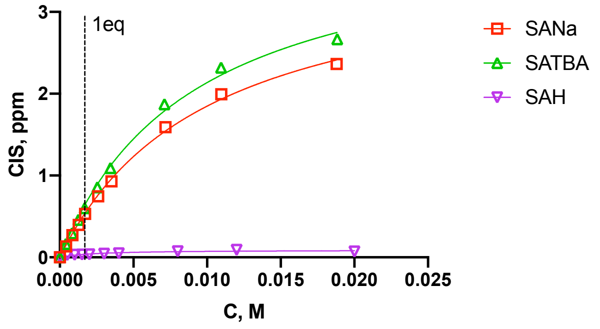

	SANa	SATBA	SAH
One site -- Specific binding [2]			No curvature
Best-fit values			
Bmax	3.699	4.073	
Ka	99.88	110.3	
Std. Error			
Bmax	0.1225	0.1793	
Ka	6.453	9.781	
95% CI (asymptotic)			
Bmax	3.416 to 3.981	3.660 to 4.487	
Ka	85.00 to 114.8	87.71 to 132.8	
Goodness of Fit			
Degrees of Freedom	8	8	
R squared	0.9981	0.9962	
Sum of Squares	0.0113	0.02985	
Sy.x	0.03759	0.06108	

Figure S1. ^{1}H NMR titration data for FM1 (urea NH(7/8) proton shift) titrated with SA·H, SA·TBA and SA·Na in DMSO-d_6 with corresponding fit of the data to a Langmuir mono-site binding model. The vertical dashed line indicates 1:1 equivalent of host and guest.

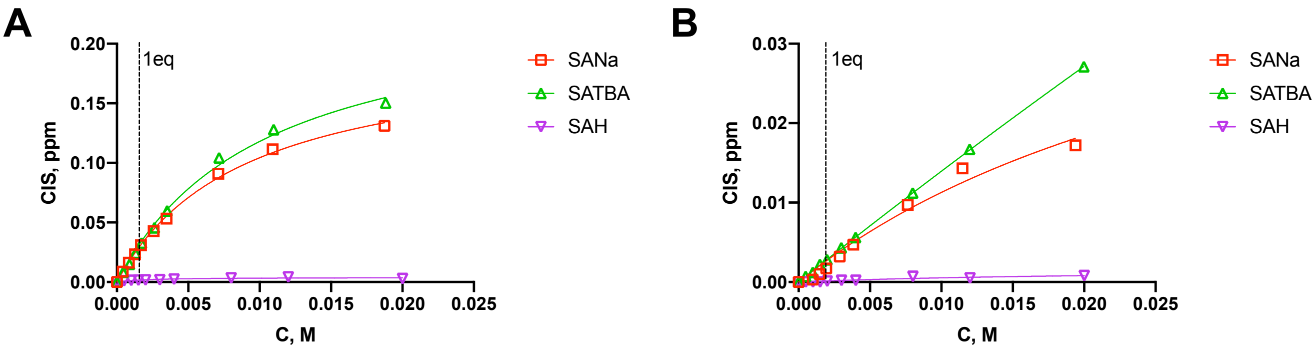

	SANa	SATBA	SAH
One site -- Specific binding [2]			No curvature
Best-fit values			
Bmax	0.1975	0.2397	
Ka	112	96.96	
Std. Error			
Bmax	0.00604	0.01276	
Ka	6.923	9.975	
95% CI (asymptotic)			
Bmax	0.1835 to 0.2114	0.2103 to 0.2691	
Ka	96.07 to 128.0	73.95 to 120.0	
Goodness of Fit			
Degrees of Freedom	8	8	
R squared	0.9981	0.9954	
Sum of Squares	0.00003469	0.0001157	
Sy.x	0.002082	0.003804	
Number of points			
# of X values	20	30	
# Y values analyzed	10	10	10

	SANa	SATBA	SAH
One site -- Specific binding [2]			No curvature
Best-fit values			
Bmax	0.05068	0.5126	
Ka	28.64	2.798	
Std. Error			
Bmax	0.01745	0.095	
Ka	13.61	0.5415	
95% CI (asymptotic)			
Bmax	0.01044 to 0.09091	0.2935 to 0.7316	
Ka	-2.757 to 60.03	1.549 to 4.046	
Goodness of Fit			
Degrees of Freedom	8	8	
R squared	0.9743	0.9999	
Sum of Squares	0.000009336	7.078E-08	
Sy.x	0.00108	0.00009406	
Number of points			
# of X values	20	30	
# Y values analyzed	10	10	10

Figure S2. ^{1}H NMR titration data for FM1 (proton shift CH(9/10)) titrated with SA·H, SA·TBA and SA·Na in DMSO-d$_6$ (A) and CD$_3$OD (B) with corresponding fit of the data to a Langmuir mono-site binding model. The data in B are unreliable due to lack of isotherm curvature. The vertical dashed line indicates 1:1 equivalent of host and guest.

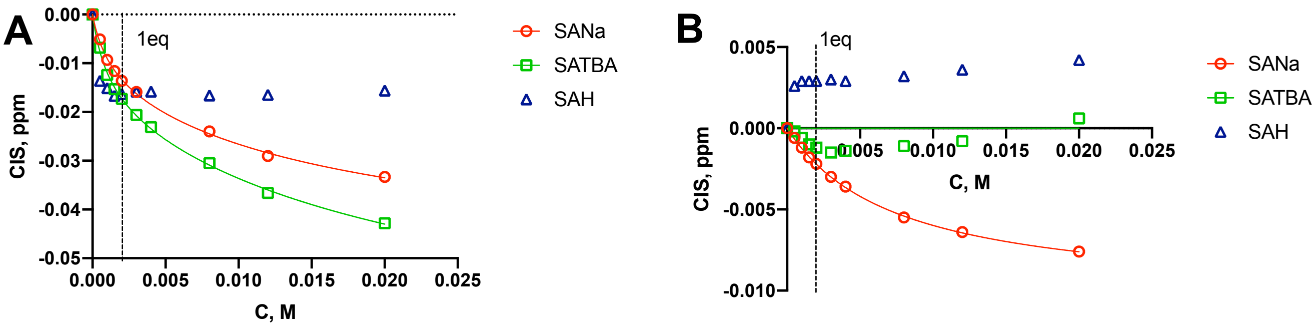

	SANa	SATBA	SA-H
Two sites -- Specific binding [2]			n/a
Best-fit values			
BmaxLo	-0.03624	-0.05297	
KaLo	53.20	40.59	
BmaxHi	-0.01568	-0.02028	
KaHi	842.8	940.6	
Percent Hi Affinity	30.20	27.68	
Std. Error			
BmaxLo	0.004702	0.009246	
KaLo	36.21	22.82	
BmaxHi	0.005948	0.004550	
KaHi	392.8	292.0	
Percent Hi Affinity	7.690	3.092	
95% CI (asymptotic)			
BmaxLo	-0.04833 to -0.02415	-0.07560 to -0.03035	
KaLo	-39.88 to 146.3	-15.25 to 96.42	
BmaxHi	-0.03097 to -0.0003861	-0.03141 to -0.009145	
KaHi	-166.9 to 1853	226.1 to 1655	
Percent Hi Affinity	10.43 to 49.96	20.12 to 35.25	
Goodness of Fit			
Degrees of Freedom	5	6	
R squared	0.9988	0.9990	
Sum of Squares	1.164e-006	1.650e-006	
Sy.x	0.0004826	0.0005244	

	SANa	SATBA	SA-H
One site -- Specific binding [2]			
Best-fit values		n/a	n/a
Bmax	-0.01044		
Ka	134.2		
Std. Error			
Bmax	0.0001125		
Ka	3.144		
95% CI (asymptotic)			
Bmax	-0.01070 to -0.01018		
Ka	127.0 to 141.5		
Goodness of Fit			
Degrees of Freedom	8		
R squared	0.9997		
Sum of Squares	1.992e-008		
Sy.x	4.989e-005		
Number of points			
# of X values	10		
# Y values analyzed	10		

Figure S3. ^{1}H NMR titration data for FM2 titrated with SA·H, SA·TBA and SA·Na in DMSO-d_6 (A) and CD_3OD (B) with corresponding fit of the data to a Langmuir binary and mono-site models. The vertical dashed line indicates 1:1 equivalents of host and guest.

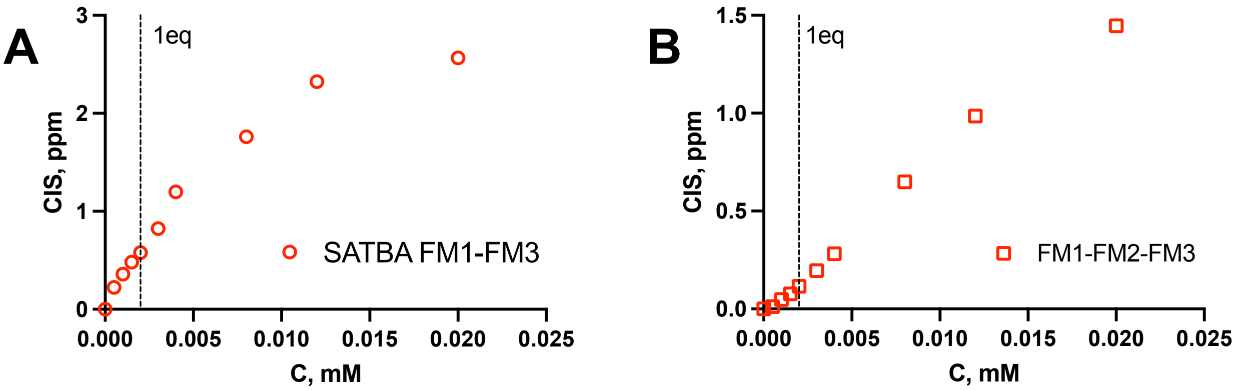

Figure S4. ^{1}H NMR titration data for FM1:FM3 (1:1) (A) and FM1:FM2:FM3 (1:1:1) (B) titrated with SA·TBA in DMSO-d$_6$. Urea NH(7/8) proton shift was monitored. The vertical dashed line indicates 1:1 equivalents of host and guest.

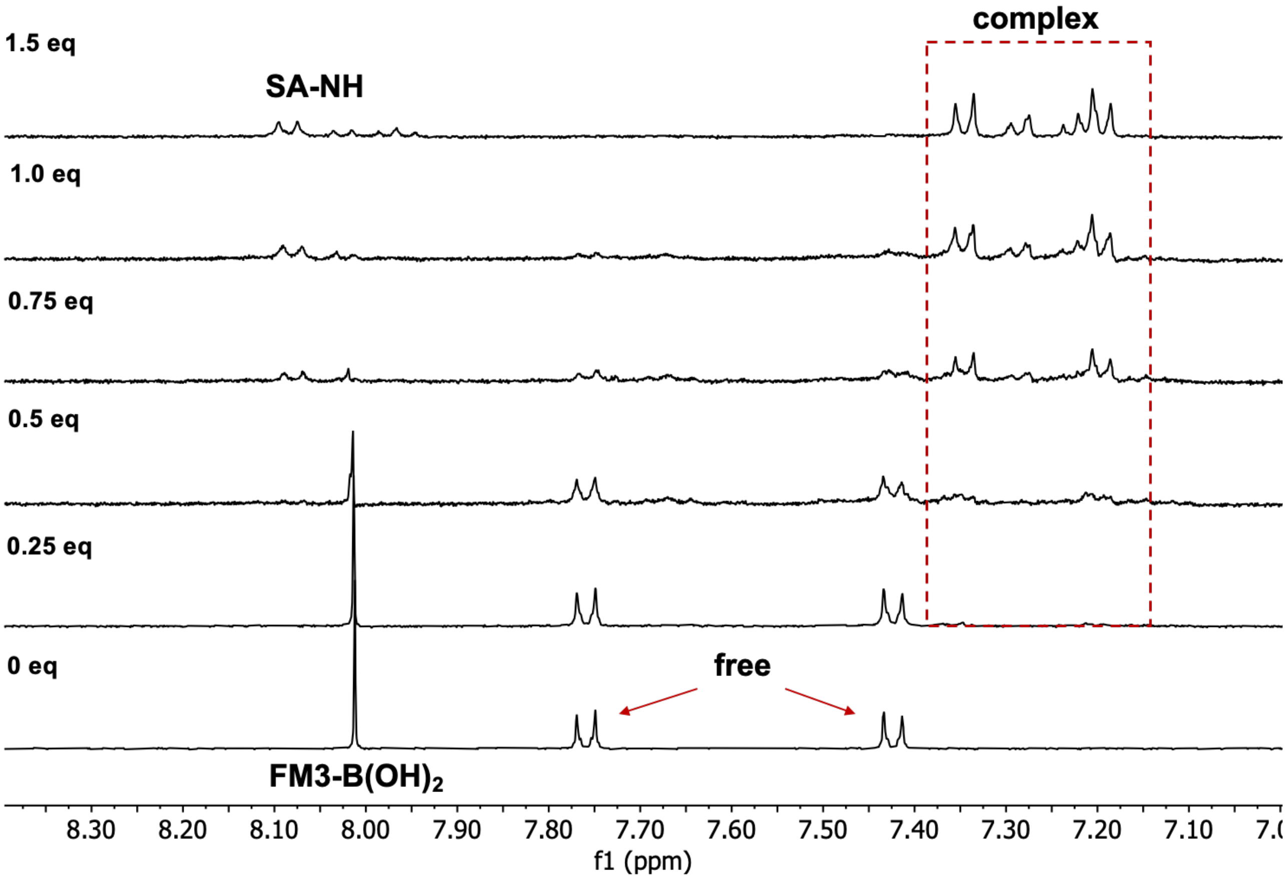

Figure S5. ^{1}H NMR titration data for 2 mM FM3 titrated with SA·TBA in DMSO-d$_6$. Aromatic region of FM3 in free and complexed form are shown.

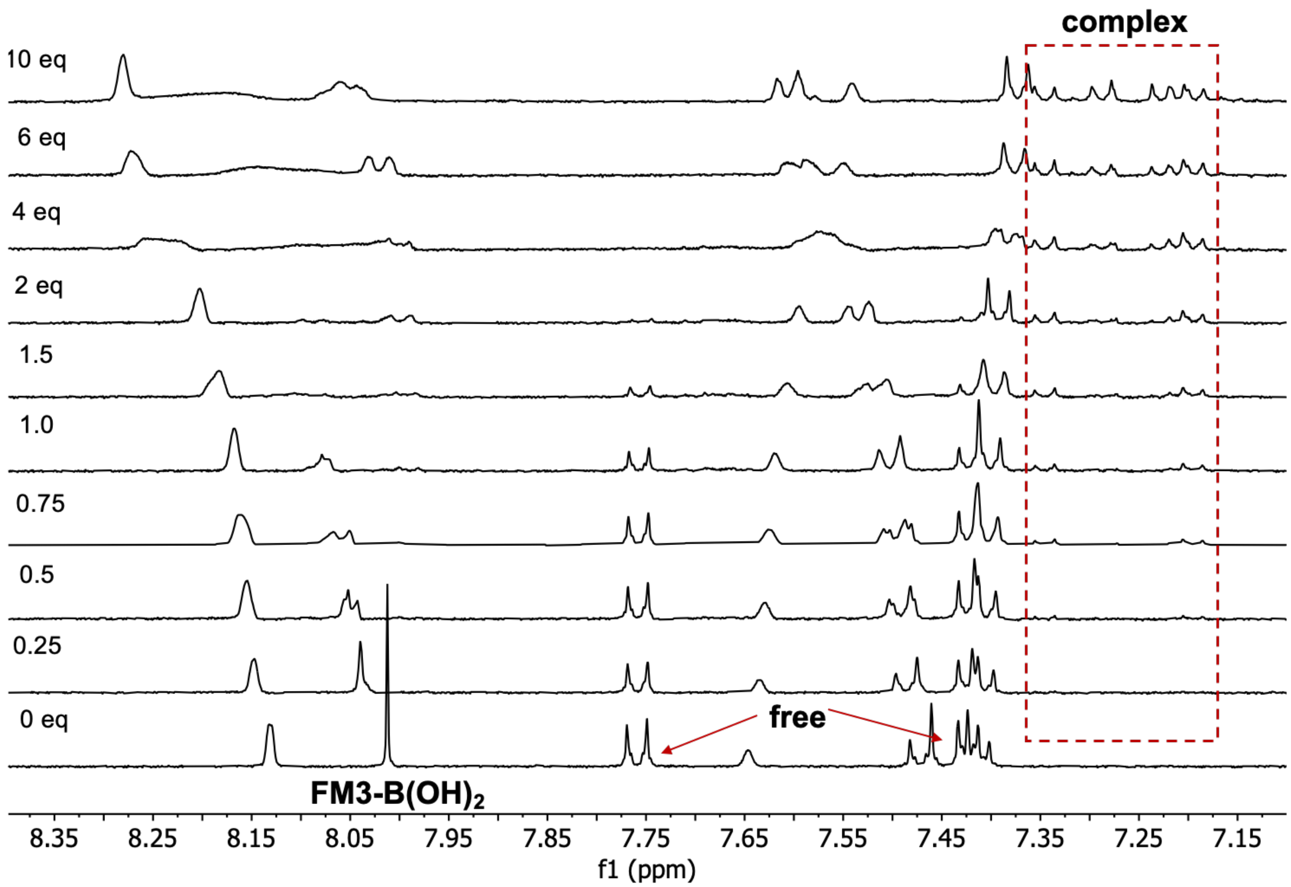

Figure S6. ^{1}H NMR titration data for 2 mM equimolar mixture of FM1 and FM3 titrated with SA·TBA in DMSO-d_6. Aromatic region of FM3 in free and complexed form are shown.

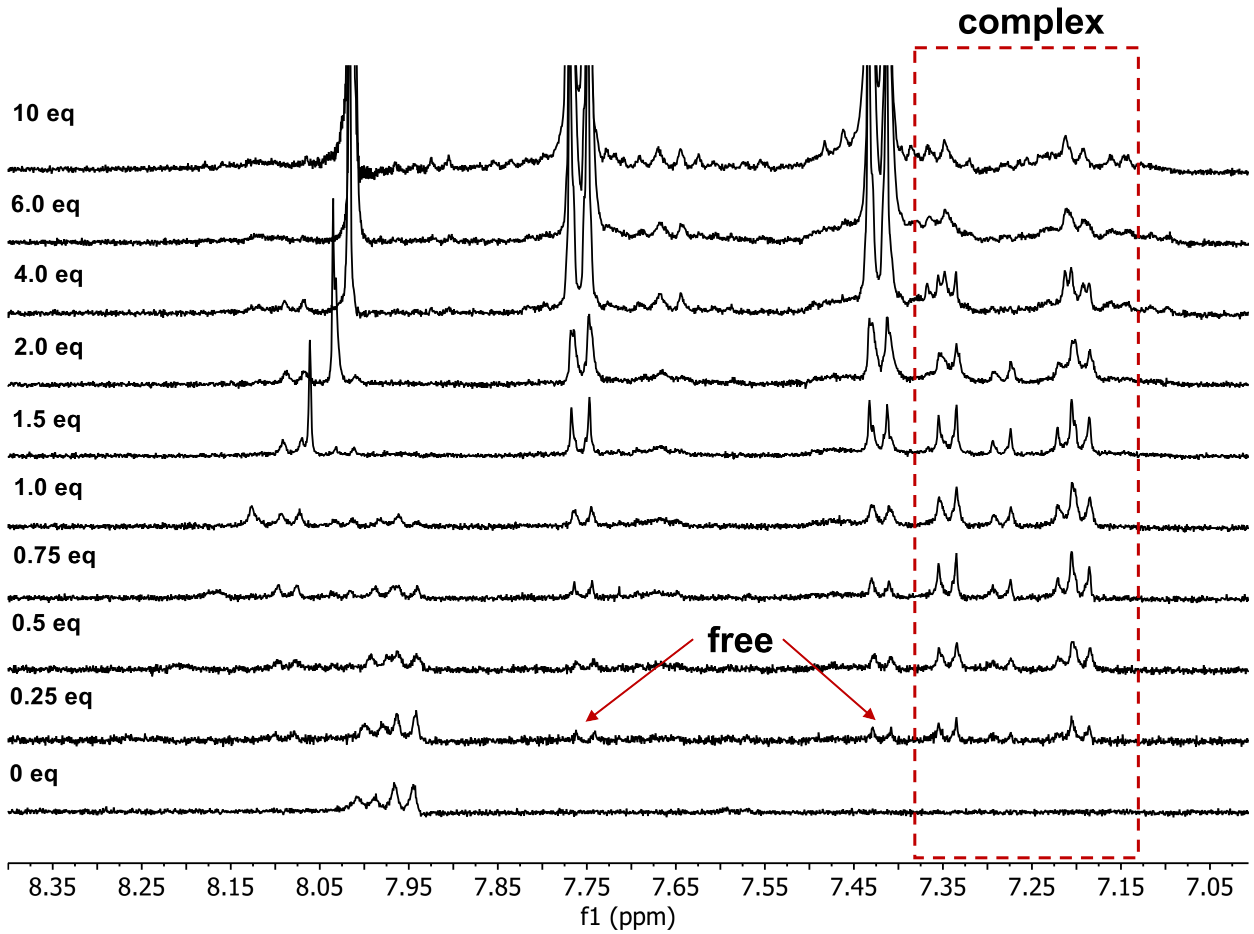

Figure S7. ^{1}H NMR titration data for 2 mM SA·TBA titrated with FM3 in DMSO-d_6. Aromatic region of FM3 in free and complexed form are shown.

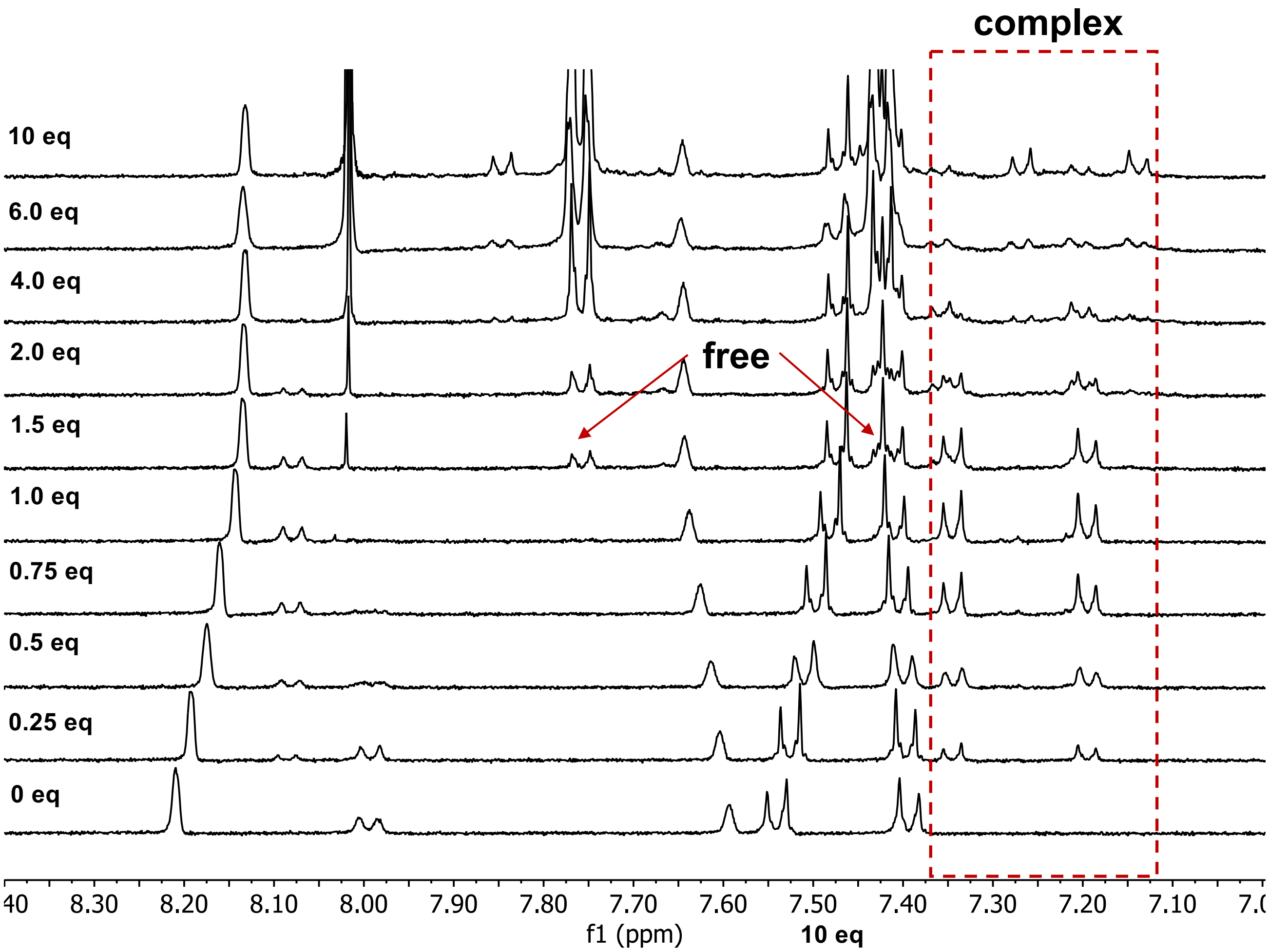

Figure S8. ^{1}H NMR titration data for 2 mM SA·TBA+FM1 (1:1) titrated with FM3 in DMSO-d_6. Aromatic region of FM3 in free and complexed form are shown.

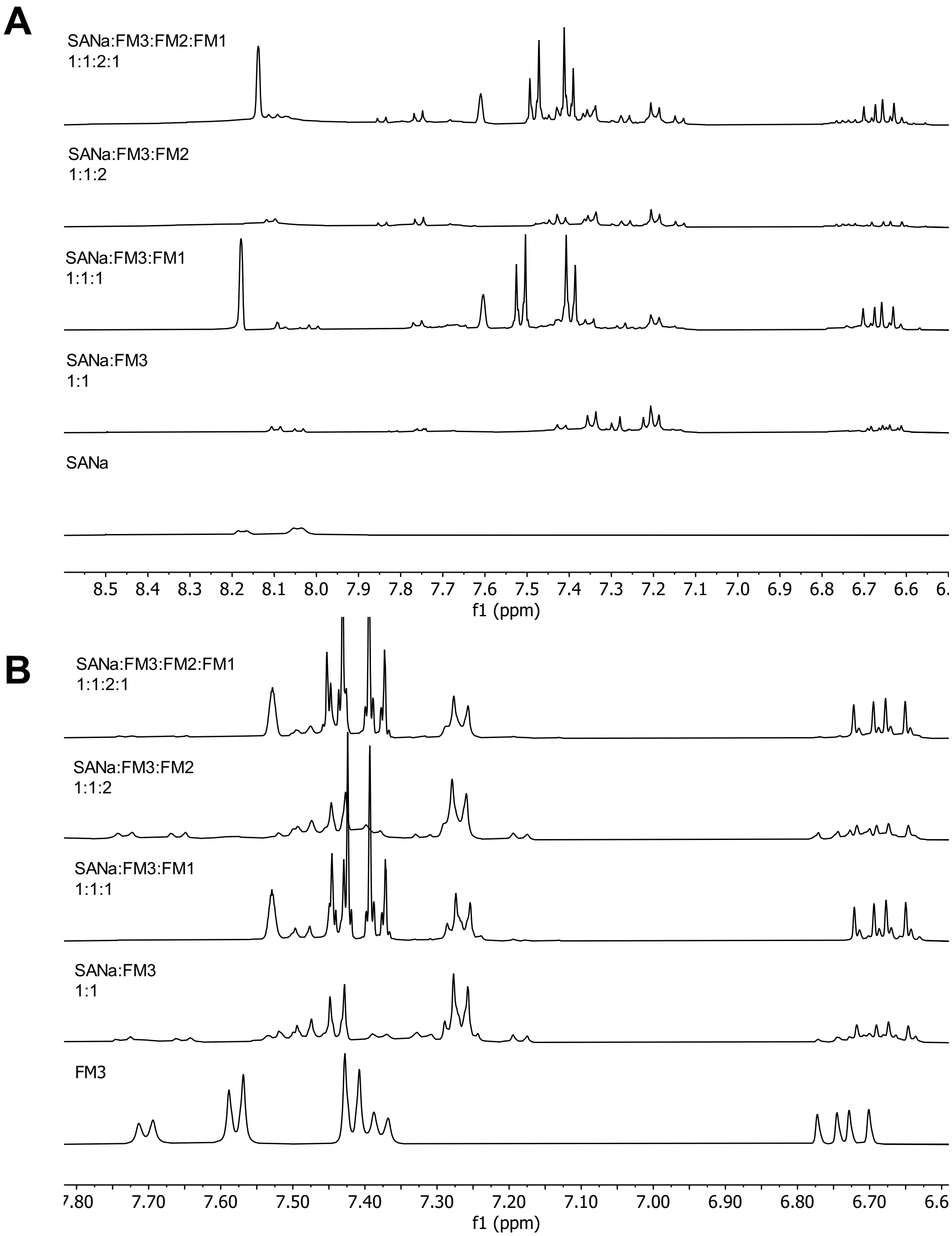

Figure S9. ^{1}H NMR titration data for 20 mM equimolar mixture of SA·Na with FM1:FM2:FM3 in DMSO-d_6 (A) and CD_3OD (B).

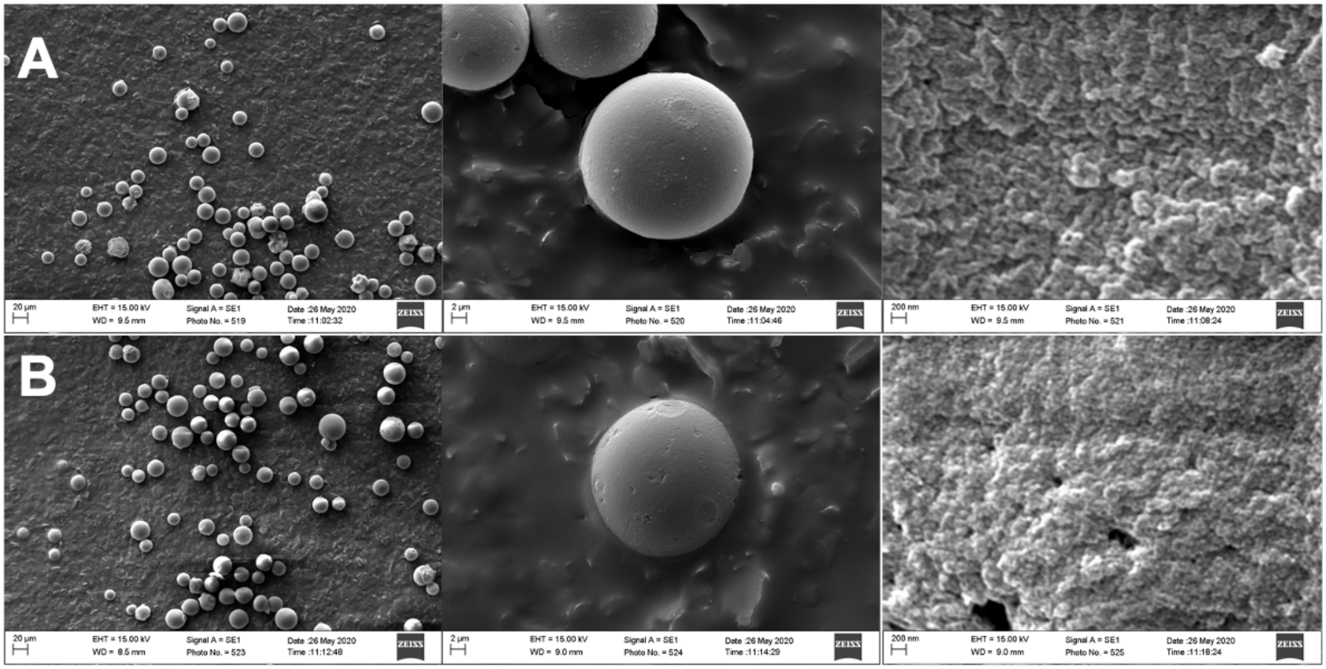

Figure S10. SEM of SA-TBA-MIP (A) and NIP (B) after silica etching recorded at 500x, 5000x and 50000 magnification.

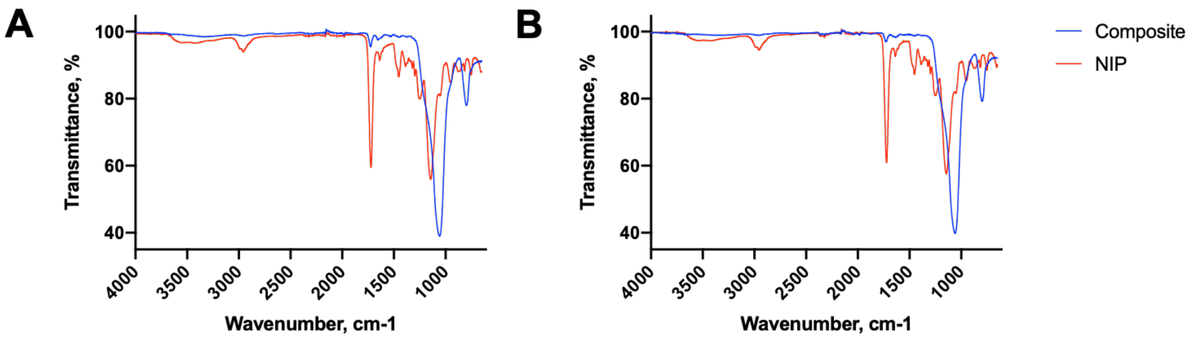

Figure S11. FTIR of the silica/polymer composite and polymer after silica etching SATBA-MIP (A) and NIP (B) showing removal of the silica scaffold and identical composition of MIP and NIP.

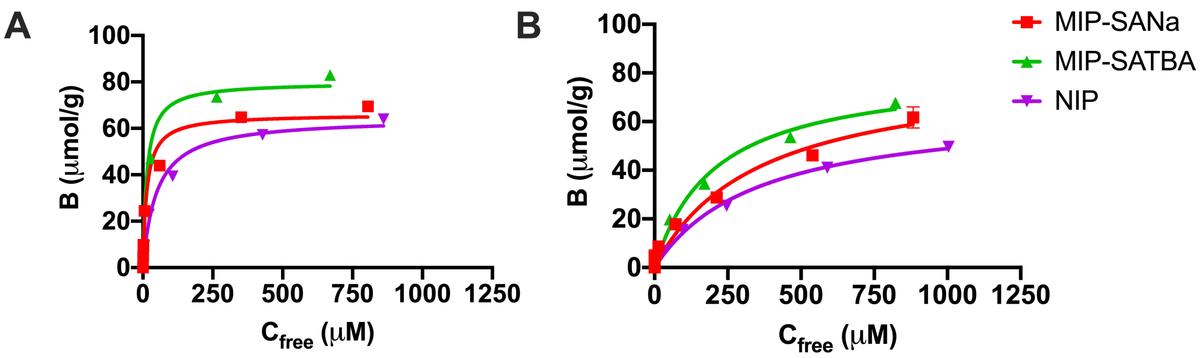

Figure S12. Binding isotherms for SA by MIP-SANa, MIP-SATBA and NIP in 100% (A) and 10% (B) methanol fitted with Langmuir binding model.

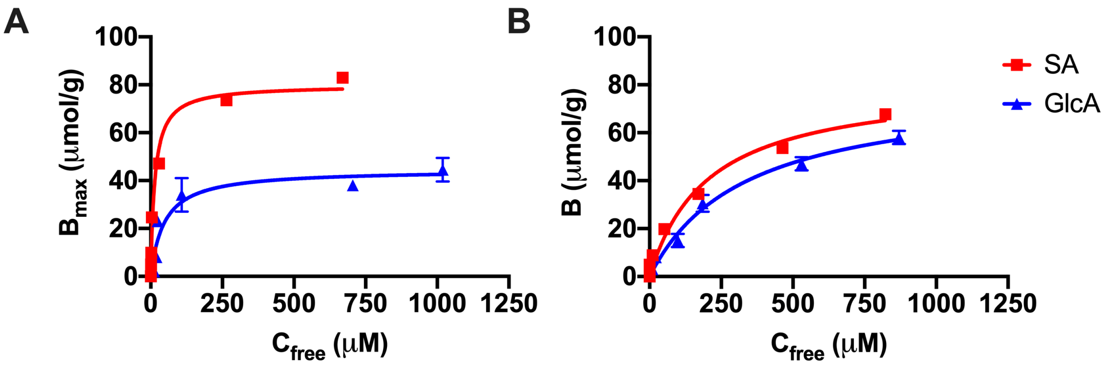

Figure S13. Binding isotherms for SATBA-MIP with SA and GlcA in 100% methanol (A) and 10% methanol (B) fitted with Langmuir binding model.
